# Spectrum of peripheral neuropathies associated with surgical interventions; A neurophysiological assessment

**DOI:** 10.1186/1749-7221-5-9

**Published:** 2010-04-19

**Authors:** Shiv Saidha, Jennifer Spillane, Gerard Mullins, Brian McNamara

**Affiliations:** 1 Department of Neurophysiology, Cork University Hospital, Cork, Ireland

## Abstract

**Background:**

We hypothesized that a wide range of surgical procedures may be complicated by neuropathies, not just in close proximity but also remote from procedural sites. The aim of this study was to classify post-operative neuropathies and the procedures associated with them.

**Methods:**

We retrospectively identified 66 patients diagnosed with post-procedure neuropathies between January 2005 and June 2008. We reviewed their referral cards and medical records for patient demographics, information on procedures, symptoms, as well as clinical and neurophysiological findings.

**Results:**

Thirty patients (45.4%) had neuropathies remote from procedural sites and 36 patients (54.5%) had neuropathies in close proximity to procedural sites. Half of the remote neuropathies (15/30) developed following relatively short procedures. In 27% of cases (8/30) remote neuropathies were bilateral. Seven patients developed neuropathies remote from operative sites following hip arthroplasties (7/30: 23.3%), making hip arthroplasty the most common procedure associated with remote neuropathies.

Sciatic neuropathies due to hip arthroplasty (12/36, 33.3%) accounted for the majority of neuropathies occurring in close proximity to operative sites.

Five medial cutaneous nerve of forearm neuropathies occurred following arterio-venous fistula (AVF) formation.

**Conclusions:**

An array of surgical procedures may be complicated by neuropathy. Almost half of post-procedure neuropathies occur remote from the site of procedure, emphasizing the need to try to prevent not just local, but also remote neuropathies. Mechanical factors and patient positioning should be considered in the prevention of post-operative neuropathies. There is a possible association between AVF formation and medial cutaneous nerve of forearm neuropathy, which requires further study for validation.

## Background

Nerve injuries are an uncommon but important complication of surgical procedures. Common mechanisms of surgery related nerve injuries include; compression, entrapment or angulation (e.g. median, ulnar and common peroneal neuropathies), traction (e.g. brachial plexopathies), direct trauma including crushing or laceration injuries, and indirect trauma (e.g. secondary to haematoma formation) [1].

Nerve injury may also occur remote from surgical sites. In such cases, it is thought that injury mostly results from patient positioning resulting in stretching and/or compression of nerves [2,3], although several factors such as abolition of protective neuromuscular responses secondary to anaesthesia are likely contributory [4].

The most commonly reported post-operative neuropathy is ulnar neuropathy, with a reported incidence of 1 in 350 [5]. Other commonly reported post-operative neuropathies include common peroneal neuropathy [6], brachial plexopathy [7,8] and radial neuropathy [9].

There are few studies of post-operative peripheral neuropathies. In this retrospective study we report a large series of post-operative nerve injuries encountered in a busy tertiary neurophysiology referral centre and aim to classify the most commonly encountered post-operative neuropathies, the procedures associated with post-operative neuropathies and provide recommendations that may help reduce the risk of these complications.

## Methods

We retrospectively identified all patients diagnosed with post-procedure neuropathies between January 2005 and June 2008 from the neurophysiology database at Cork University Hospital. Cork University Hospital is a large tertiary referral hospital in the southwest of Ireland with a catchment population of 1.5 million. It is the only centre providing clinical neurophysiology in this region, affording us the unique opportunity to perform epidemiological studies.

All referred patients were assessed by a consultant clinical neurophysiologist (BMN) and underwent nerve conduction studies and electromyography (EMG). Patients with upper limb symptoms had sensory nerve conduction studies of the median, ulnar and radial nerves, as well as motor studies of the median and ulnar nerves, both on the symptomatic and asymptomatic side. Medial cutaneous and lateral cutaneous nerves of forearm were studied in patients with forearm sensory symptoms and patients with suspected brachial plexopathy. Needle EMG was performed to assess the degree of denervation in patients with radial and ulnar neuropathies and to aid accurate diagnosis of brachial plexopathy.

All patients with lower limb symptoms had sensory nerve conduction studies of the sural and superficial peroneal nerves bilaterally. Patients with sensory symptoms in the thighs also had bilateral sensory nerve conduction studies of the lateral femoral cutaneous nerves. Motor conduction studies of the common peroneal nerves and tibial nerves were performed bilaterally. Patients with lower limb weakness had extensive needle EMG of the lower extremities to determine the degree (prognosis) and distribution (localize lesion) of denervation.

Information on patient symptoms, interval between surgical procedure and symptom-onset, type of procedure and reason for procedure were obtained from patient referral cards and their medical records. Demographic information, medical history and medication history were also collected in each case. Local ethical approval was obtained to allow review of patient notes.

Patients with pre-operative symptoms and patients with inconclusive, normal or neurophysiology not in keeping with symptoms were excluded from further analysis. Only patients whose symptoms started within one week of surgery were included in the study. Patient notes were extensively reviewed to determine if surgery or other post operative factors were the likely cause of neuropathy. In the latter case, those patients were excluded from further analysis.

## Results

Eighty-two patients with post-procedure neuropathies were identified, of which 66 fulfilled the study inclusion criteria (Age range: 16-84, Average age: 56, Female: 28, Male: 38). Thirty patients (45.4%) had neuropathy remote from the site of procedure (Additional File 1) and 36 patients (54.5%) had neuropathy in close proximity to the site of procedure (Additional File 2). Patient co-morbidities, relevant medication exposure, as well as clinical and neurophysiological follow-up data are also included in Additional Files 1 &2.

Ulnar (6/30: 20%), lateral cutaneous nerve of thigh (5/30: 16.6%), common peroneal (4/30: 13.3%), median (3/30: 10%), sciatic (3/30: 10%) and femoral (3/30: 10%) neuropathies were the most frequent neuropathies remote from the procedural site. Eight patients (8/30: 27%) had neuropathies remote from the procedural site which were bilateral; 3 ulnar, 2 lateral cutaneous nerve of thigh, 1 median, 1 femoral and 1 lumbrosacral plexopathy.

**Figure 1:**
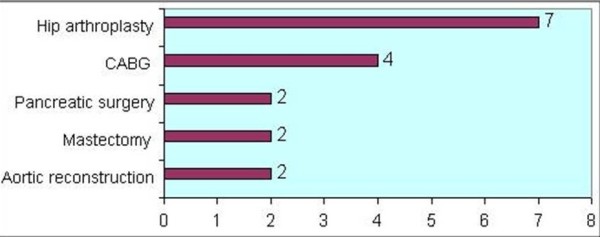
**Procedures most commonly associated with remote neuropathy**.

7 patients developed neuropathies (3 ulnar, 2 lumbrosacral plexus, 1 radial, 1 lateral cutaneous nerve of thigh) remote from the operative site following hip arthroplasties (7/30: 23.3%), making hip arthroplasty the most common type of surgical procedure associated with remote neuropathies (Procedures most commonly associated with remote neuropathies are illustrated in Figure 1). Post hip arthroplasty neuropathies were bilateral in 3 patients.

All neuropathies that developed following gastro-intestinal (6; Additional File 3), cardio-thoracic (6; Additional File 3), obstetric & gynaecological (2; Additional File 3) and breast (2; Additional File 3) surgery occurred remote from operative sites.

Of the 6 neuropathies occurring following cardiothoracic surgery, 4 developed after coronary artery by-pass grafting and 2 following ascending aorta reconstruction.

Bilateral ulnar neuropathies developed following L5 discectomy and spinal plasmacytoma decompression procedures (Additional File 3). Other procedures associated with remote neuropathies included; colonoscopy (1 common peroneal neuropathy), prostatectomy (1 femoral neuropathy) and coronary angiography (1 median neuropathy contralateral to the side of arterial cannulation).

Sciatic neuropathies due to hip arthroplasty (12/36: 33.3%) accounted for the majority of neuropathies occurring in close proximity to the operative site. These were fascicular (predominantly affecting the common peroneal fascicle of the sciatic nerve) in 7 (7/12: 58.3%).

All neuropathies following vascular surgery occurred in close proximity to the site of surgical procedure (12/36: 33.3%). Arterio-venous fistula (AVF) formation was associated with the majority of post vascular surgery neuropathies (6/12: 50%), and 5 medial cutaneous nerve of forearm neuropathies developed following AVF formation (5/6: 83.3%).

## Discussion

Clinical neurophysiological assessment of patients with suspected post-operative nerve injuries not only allows diagnosis of the anatomical nerve lesion, but also classification of the type of nerve injury in order to guide further management. According to the Seddon classification, nerve injuries can be classified into 3 categories; neuropraxia (mild: focal demyelination after focal injury; axon intact), axonotemesis (moderate: axonal loss; nerve sheath intact) and neurotemesis (severe: axonal loss and disruption of nerve sheath) [10]. Patients with neuropraxia have a favourable outcome, while patients with neurotemesis have a poor prognosis if untreated (i.e. failure to surgically repair the damaged nerve).

Post-procedure neuropathy is a clinically important and probably under-recognised/diagnosed entity. An American society of Anaesthesiologists closed claims study previously showed that 15% of all claims related to nerve injury [11]. Although aetiological mechanisms of post-procedure neuropathies are well described, such injuries may result in litigation for pain, morbidity and economic consequences [12,13]. Neuropathy occurring in close proximity to the site of procedure is mostly due to direct neural trauma i.e. by needles, instruments, diathermy, local injections, ischaemia and suturing [14]. Mechanical factors such as patient positioning, pressure at the operative site [2,3] and stretching of nerves during procedures are also likely contributory [15].

In this study sciatic neuropathy following hip arthroplasty and medial cutaneous nerve of forearm neuropathy following AVF formation were the most common surgery specific close proximity neuropathies. The association between sciatic neuropathy and hip arthroplasty is well established [16-19]. Given the risk of sciatic nerve injury during hip arthroplasty, some centres now routinely monitor for potential nerve injury with intra-operative monitoring using evoked potentials and free run EMG to warn surgeons of potential peripheral nerve damage during surgery [20]. The association between AVF formation and medial cutaneous nerve of forearm neuropathy is poorly recognised. Perhaps a contributing risk factor for the development of neuropathy in these patients in this study was underlying chronic renal failure, as all patients having AVFs formed were for renal dialysis therapy. A larger study of neuropathy following AVF formation may help validate this association.

Half of the neuropathies which occurred remote from procedural sites in this study developed following relatively short procedures, during which one would expect limited opportunity for clinically relevant nerve compression to occur, raising the possibility that there may have been additional contributory factors. While careful patient positioning and application of padding to sites of pressure/compression such as the elbows may help reduce the risk of developing neuropathies, there is no such study confirming this.

Apart from direct neural trauma and mechanical factors, other factors may also contribute to the development of post-procedure neuropathy. Nerves may be more susceptible to trauma as a result of pre-existing generalised peripheral neuropathy [9,21], local compression neuropathy (overt or subclinical) [4,22] or as a hereditary predisposition (hereditary neuropathy with liability to pressure palsy/HNPP) [23,24]. HNPP should be considered in patients with post-operative focal neuropathies. It often has typical neurophysiological findings and was considered in our population. Only one patient in this study had neurophysiological features to support HNPP, and genetic testing was not performed as the patient declined this.

Procedure duration appears to contribute to the development of post-operative neuropathy, with all neuropathies observed following gastro-intestinal, cardio-thoracic and breast surgery being remote from operative sites. Increased procedure duration may allow prolonged periods of nerve compression, increasing the risk for development of neuropathy.

The number of post-procedure neuropathies in this study is likely under-representative. Possible reasons for this include; under-reporting, under referral for clinical neurophysiology assessment, the belief that these injuries will be self-limiting or that these injuries appear minor compared to the underlying problem requiring intervention.

A wide variety of surgical procedures may be complicated by neuropathy, both in close proximity and remote from operative sites. The aetiology of post-procedure neuropathies appears multi-factorial. Preventative measures have been outlined above and vary from simple careful positioning to complex intra-operative neurophysiological monitoring depending on the procedure undertaken. Almost half of all post-procedure neuropathies occur remote from the site of procedure, emphasizing the need to prevent not just local, but also remote neuropathies. There may be an association between AVF formation and medial cutaneous nerve of forearm neuropathy, which requires further study for validation.

## Competing interests

The authors declare that they have no competing interests.

## Authors' contributions

SS was involved in the conception of the study, study design and co-ordination, review of medical records, data gathering and analysis. JS was involved in the review of medical records, data gathering and analysis. GM was involved in the study design and co-ordination, data gathering and analysis. BMN was involved in the conception of the study, study design and co-ordination, review of medical records, data gathering and analysis. All authors read and approved the final manuscript.

## Supplementary Material

Additional file 1 **Table 1**. Contains a table detailing information regarding neuropathies which occurred remote from the site of procedure including relevant co-morbidities, relevant medication exposure, major neurophysiological findings at initial assessment and follow-up clinical and neurophysiological findings 6-12 months later. Click here for file

Additional file 2 **Table 2**. Contains a table detailing information regarding neuropathies which occurred in close proximity to the site of procedure including relevant co-morbidities, relevant medication exposure, major neurophysiological findings at initial assessment and follow-up clinical and neurophysiological findings 6-12 months later. Click here for file

Additional file 3 **Table 3**. Contains a table illustrating neuropathies associated with specific types of surgery. Click here for file
